# Association between ABO blood groups and hematological myeloid neoplasms in adolescents and adults

**DOI:** 10.3389/fmed.2024.1456695

**Published:** 2024-09-17

**Authors:** Miao Zhou, Tongyu Li, Yongcheng Sun, Guifang Ouyang, Wanchuan Zhuang, Ping Zhang

**Affiliations:** ^1^Department of Hematology, The First Affiliated Hospital of Ningbo University, Ningbo, Zhejiang, China; ^2^Department of Hematology, Lianyungang Second People’s Hospital, Lianyungang, Jiangsu, China

**Keywords:** ABO blood groups, hematological myeloid neoplasms, adolescents, adults, association

## Abstract

**Background:**

Prior research suggests a potential link between ABO blood types and susceptibility to various malignancies. The correlation between ABO blood types and hematological myeloid neoplasms, however, remains inadequately explored.

**Objective:**

This study investigates the association between ABO blood groups and the incidence of hematological myeloid neoplasms in adolescents and adults.

**Methods:**

In this retrospective clinical study, 1,022 adolescent and adult cases of myeloid neoplasms diagnosed at our institution were initially considered. After excluding conditions potentially linked to ABO blood types from prior studies, 792 eligible cases were analyzed. These cases were categorized based on disease subtypes and compared with a control group for blood type distribution.

**Results:**

Our findings reveal a significantly higher prevalence of blood type A in patients with myeloid neoplasms compared to the control group, except for chronic myelocytic leukemia and myeloproliferative neoplasms. Conversely, the prevalence of blood type AB in myeloid neoplasms was notably lower than in the control group.

**Conclusion:**

The study suggests a potential association between ABO blood types and the risk of developing hematological myeloid neoplasms in adolescents and adults. Further research is warranted to elucidate the underlying mechanisms of this relationship.

## 1 Introduction

The ABO blood group system, a focus of scientific inquiry for over a century, is governed by specific glycosyltransferase genes located on chromosomes 9q34.1 to 9q34.2 (1). This system, beyond its fundamental role in transfusion medicine, has been implicated in various health conditions, including endocrine and metabolic disorders, cardiovascular diseases, and more recently, oncogenesis (2, 3). Alterations at the molecular level in ABH antigen glycosylates have been shown to modify antigen structure and quantity, affecting cell adhesion and signaling pathways, and potentially contributing to tumor development and progression (4).

In recent clinical observations, there appears to be an uptick in patients presenting with hematologic malignancies. This trend could be attributed to heightened healthcare awareness and advancements in diagnostic techniques. Hematologic malignancies, primarily rooted in cellular and molecular genetic abnormalities, often present with obscure risk factors, unlike some solid tumors. These malignancies are possibly linked to environmental influences, chemical exposure, radiation, and genetic predispositions. However, in clinical practice, patients with a definitive genetic or exposure history represent only a small fraction of the broader hematologic disease population. Identifying epidemiological factors associated with these malignancies, particularly those that are readily assessable, could significantly contribute to public health education and early detection strategies.

Previous studies, notably in 2004 and 2006, have explored the correlation between pediatric hematological malignancies and ABO blood groups (5, 6). These studies focused on children under 12 years of age and yielded inconsistent conclusions among overlapping disease categories. Since then, research in this area has been scant. Our retrospective study, therefore, fills a gap in current knowledge by specifically examining the relationship between ABO blood groups and adult hematological myeloid neoplasms. To refine our research, we meticulously excluded cases with potential confounding diseases related to blood type. This approach is novel, as no similar studies focusing on this adolescent and adult demographic have been reported to date.

## 2 Methodology

This retrospective study analyzed 1,022 adolescent and adult cases (aged 16–92 years) of myeloid neoplasms, including acute myeloid leukemia (AML), chronic myeloid leukemia (CML), myelodysplastic syndromes (MDS), and chronic myeloproliferative neoplasms (MPN), diagnosed at the First Affiliated Hospital of Ningbo University between 1 October 2017, and 31 October 2023. Diagnostic criteria were based on cell morphology, immunophenotyping, and relevant molecular and cytogenetic changes, adhering to the 2016 WHO classification criteria for hematopoietic and lymphoid tumors (7).

Exclusion criteria encompassed factors like adverse environmental and substance exposures, clear familial tumor history, concurrent malignant tumors, hepatitis B virus infection, type 2 diabetes, coronary heart disease, myocardial infarction, deep vein thrombosis, hypertension, hyperlipidemia, tuberculosis, and HIV infection (2, 3, 8–14). Of the initial cohort, 792 patients met the study criteria, with only four RH-negative cases; the rest were RH-positive.

For the control group’s blood group distribution, due to the lack of official data, we referenced the 2018 study by Liu et al. (15), which conducted a comprehensive blood group census of 3,827,125 Chinese adults. This study utilized data from the NFPHE, a nationwide physical check-up program across 31 provinces in China targeting married couples aged 21–49 years who were preparing for pregnancy. It represented the most extensive population-based data available to date.

The analysis was performed using SPSS version 25.0. The overall distribution of different blood groups across the AML, CML, MDS, and MPN cohorts was compared with that of the control group using the Chi-square test. The 95% confidence interval (CI) was calculated using binomial probability. To account for the increased risk of Type I error due to multiple comparisons, Bonferroni correction was applied to the *P*-values, yielding a corrected *P*_*c*_-value. Any *P*_*c*_-value greater than 1 was represented as “–.”

## 3 Results

### 3.1 General patient characteristics

**FIGURE 1:**
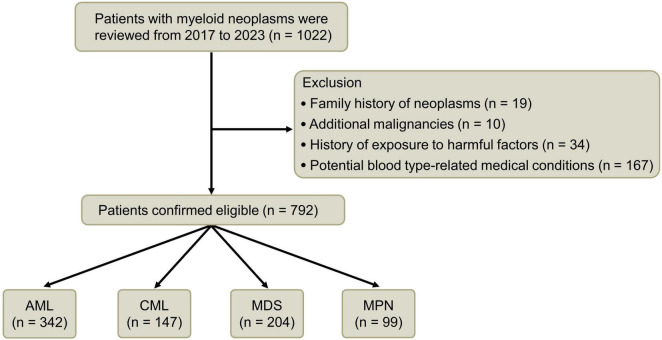
Flowchart illustrating the cohort selection process.

Following a meticulous screening process, a comprehensive cohort of 792 patients was included in the clinical data investigation, comprising 463 individuals of the male gender and 329 individuals of the female gender. The median age was 63 (16–92) years. Within this cohort, there were 342 cases diagnosed with AML, 147 patients afflicted by CML, 204 individuals experiencing MDS, and 99 patients diagnosed with MPN (Figure 1 and Table 1).

**TABLE 1:** General characteristics of enrolled patients.

Characteristic data	Number of cases (%)
**Sex**	
Male	463 (58.5%)
Female	329 (41.5%)
**Body mass index (kg/m^2^)**	
< 18.5	44 (5.6%)
18.5–23.9	535 (67.5%)
≥ 24	213 (26.9%)
**Level of education**	
Primary school or below	172 (21.7%)
Secondary school	416 (52.5%)
College or above	204 (25.8%)
**Smoking status**	
Never smokers	403 (50.9%)
Former smokers	197 (24.9%)
Current smokers	192 (24.2%)
**Alcohol drinking status**	
Non-drinkers	284 (35.9%)
Drinkers	401 (50.6%)
Among drinkers	107 (13.5%)
**ECOG PS**	
≤ 2	420 (53%)
> 2	372 (47%)

### 3.2 Distribution of ABO blood groups among AML patients

Among the 342 individuals diagnosed with AML, the prevalence of blood type A stood at 41.52% (142/342; Table 2), a figure that exhibited a noteworthy elevation compared to the control cohort (30.54%; *P*_*c*_ < 0.05). In contrast, the occurrence of the AB blood group within the AML patient population was 4.39% (15/342), marking a considerable reduction in contrast to the control group (9.66%; *P*_*c*_ < 0.05). The proportion of individuals possessing blood type O among AML patients was 26.02% (89/342), which was slightly lower than that observed in the control group (30.37%), albeit without statistical significance (*P*_*c*_ > 0.05). Furthermore, the representation of blood type B within the AML patient cohort (96/342, 28.07%) closely mirrored that of the control group (29.42%), with no statistically significant disparity evident (*P*_*c*_ > 0.05).

**TABLE 2:** Comparative analysis of ABO blood group distribution and differences in AML Patients.

Blood group	Expected percentage %	Patients		95% CI	*P*-value	*P*_*c*_-value
		Number	Percentage %			
A	30.54	142	41.52	1.30–2.00	0.000010	0.000040*
B	29.42	96	28.07	0.74–1.12	0.582707	–
O	30.37	89	26.02	0.63–1.03	0.080292	0.321168
AB	9.66	15	4.39	0.26–0.72	0.000953	0.003812*
Total		342	100.0	–	–	–

*P_*c*_ < 0.05.

### 3.3 Distribution of ABO blood groups among CML patients

Within the cohort of 147 patients afflicted by CML, the prevalence of blood type AB was noted at 3.4% (5/147; Table 3), which demonstrated a marked reduction in comparison to the control group (9.66%; *P*_*c*_ < 0.05). The occurrence of blood group A in CML patients was recorded at 38.78% (57/147), representing a elevation when contrasted with the control group but the difference is not significant (*P* < 0.05, *P*_*c*_ > 0.05). Blood types B and O were observed in the CML patient population at proportions of 29.25% (43/147) and 28.57% (42/147), respectively. This aligns closely with the distribution of blood type in the control group (29.42% for type B and 30.37% for type O) and is not statistically significant (*P*_*c*_ > 0.05).

**TABLE 3:** Comparative analysis of ABO blood group distribution and differences in CML patients.

Blood group	Expected percentage %	Patients		95% CI	*P*-value	*P*_*c*_-value
		Number	Percentage %			
A	30.54	57	38.78	1.03–2.01	0.030127	0.120508
B	29.42	43	29.25	0.70–1.42	0.963411	–
O	30.37	42	28.57	0.64–1.31	0.634853	–
AB	9.66	5	3.40	0.14–0.80	0.010167	0.040668*
Total		147	100.0	–	–	–

*P_*c*_ < 0.05.

### 3.4 Distribution of ABO blood groups among MDS patients

Within the cohort of 204 individuals diagnosed with MDS, the prevalence of blood type A was notably elevated at 45.10% (92/204; Table 4), exhibiting a statistically significant difference when compared to the control group’s A blood group distribution (30.54%; *P*_*c*_ < 0.05). In contrast, the occurrence of the AB blood group among MDS patients was limited to 2.94% (6/204), representing a significant decrease relative to the control group’s distribution (9.66%; *P*_*c*_ < 0.05). Blood type O was observed in 23.53% (48/204) of MDS patients, which was lower than the normal control group’s frequency (30.37%), but not statistically significant (*P* < 0.05, *P*_*c*_ > 0.05). Furthermore, the representation of blood type B within the MDS patient cohort (58/204, 28.43%) closely paralleled that of the control group (29.42%), with no statistically significant variation evident (*P* > 0.05).

**TABLE 4:** Comparative analysis of ABO blood group distribution and differences in MDS patients.

Blood group	Expected percentage %	Patients		95% CI	*P*-value	*P*_*c*_-value
		Number	Percentage %			
A	30.54	92	45.10	1.42–2.46	0.000006	0.000024*
B	29.42	58	28.43	0.70–1.29	0.755690	–
O	30.37	48	23.53	0.51–0.98	0.033553	0.134212
AB	9.66	6	2.94	0.13–0.64	0.001154	0.004616*
Total		204	100.0	–	–	–

*P_*c*_ < 0.05.

### 3.5 Distribution of ABO blood groups among MPN patients

Among the cohort of 99 patients diagnosed with MPN, the prevalence of blood type A stood at 38.38% (38/99; Table 5), whereas the control group exhibited a distribution of blood type A at 30.54%. Blood type B was found in 30.30% (30/99) of MPN patients, while the control group displayed a comparable frequency of 29.42%. Additionally, 30.30% (30/99) of MPN patients had blood type O, a proportion that did not exhibit any statistically significant distinction from the control group for blood types A, B, and O, all of which exceeded 30.37% (*P*_*c*_ > 0.05). Conversely, the presence of the AB blood group within MPN patients was minimal at 1.01% (1/99), representing a significant decrease in contrast to the control group’s distribution (9.66%; *P*_*c*_ < 0.05).

**TABLE 5:** Comparative analysis of ABO blood group distribution and differences in MPN patients.

Blood group	Expected percentage %	Patients		95% CI	*P*-value	*P*_*c*_-value
		Number	Percentage %			
A	30.54	38	38.38	0.95–2.13	0.090092	0.360368
B	29.42	30	30.30	0.68–1.60	0.847821	–
O	30.37	30	30.30	0.65–1.53	0.987965	–
AB	9.66	1	1.01	0.01–0.68	0.006064	0.024256*
Total		99	100.0	–	–	–

*P_*c*_ < 0.05.

## 4 Discussion

The ABO blood type is characterized by the carbohydrate component present on the exterior of red blood cells, which is linked to a protein framework known as the H antigen. Beyond their presence on the surface of red blood cells, ABO antigens are also prominently found on the surfaces of epithelial cells within the gastrointestinal tract, urogenital tract, and respiratory alveolar epithelium (16). Consequently, their significance extends far beyond the realms of blood transfusions and organ transplants. ABH antigens are primarily carbohydrates found on glycoproteins, and alterations in surface glycoconjugation can result in modifications to intercellular adhesion, cell membrane signaling, and immune surveillance, potentially exerting a significant influence on cancer development (17).

In a study by Marionneau et al. (18), the A blood group gene was transfected into mice with colon cancer, resulting in reduced apoptosis of all cells expressing the A antigen and a rapid tumor growth compared to the control group. This suggests a potential relationship between ABO blood group antigens and tumor aggressiveness and prognosis. Yamamoto et al’s (19) research also indicated that ABH antigen expression can change during the formation, onset, and progression of tumors. ABO promoter methylation analysis revealed high methylation levels in various types of tumor cell lines and clinical patient specimens (20). These molecular biological mechanisms at the cellular level provide the foundation for understanding the association between ABO blood groups and clinical diseases. Furthermore, studies have reported the dominance distribution of various malignant tumors according to blood group, such as pancreatic cancer (19), gastric cancer (21), nasopharyngeal cancer (10), and others.

Abouzari and his team conducted two separate retrospective studies using the same healthy control population, one in 2008 and another in 2012. The results demonstrated that the proportion of type A blood in patients with primary and secondary central nervous system lymphoma was significantly low, while the proportion of type B blood was increased (22, 23). Recent research, including a 2017 study by Huang et al. (24) has also explored the connection between blood types and tumor development. They found that genetic traits related to ABO blood groups may influence the development of gastrointestinal and urinary tract cancers. However, no significant associations were found between blood types and the risk of sarcoma, lymphoma, leukemia, or other types of cancer. It is worth noting that the authors acknowledge that studies on leukemia and pancreatic cancer had relatively small sample sizes, which may have affected the precision of their results. The relationship between ABO blood groups distribution and malignant hematological diseases remains controversial. Recently, some scholars have pointed out that ABO blood type is related to the response of CML patients to imatinib (25).

During the retrospective 5-year period of this study, a total of 1,022 patients with myeloid neoplasms were diagnosed in our department. To investigate the potential advantage of specific blood groups in myeloid neoplasms and enhance the accuracy of our comparative analysis, we carefully reviewed existing literature and excluded other coexisting diseases that have previously been reported to have associations with blood group distribution advantages. These excluded conditions encompassed various types of malignant tumors, thrombotic diseases, coronary heart disease, and others. It is worth noting that these diseases have been documented to have correlations with specific blood types, and indeed there were cases with such combinations in our center. Additionally, it is important to note that Liu et al.’s (15) study included participants with hepatitis B, which was an exclusion criterion in our study. However, given the robust methodology used to generate the control cohort in Liu et al.’s (15) research, we still consider their data to be a relatively reliable control for comparison. As a result, a total of 792 cases were ultimately included in our final comparative study.

Our findings revealed a notable decrease in the proportion of blood type AB among all disease groups when compared to the control group. Conversely, patients with blood type A displayed a consistently higher proportion in myeloid neoplasms, except for those with CML and MPN. It is important to note that in MPN patients, the low count of individuals with blood type AB prevented us from considering it as conclusive evidence. However, we also observed a lower proportion of blood type AB and a higher proportion of blood type A in AML and MDS patients. In fact, since almost all AML patients and a significant portion of MDS patients undergo complete blood type testing as part of their treatment, we can confidently establish statistical differences in blood types among AML and MDS patients. Conversely, most patients with CML and MPN do not require blood type collection during the diagnosis and treatment process, which could be a significant factor influencing the accuracy of blood type data for these particular diseases.

Regarding the blood type “susceptibility,” Garratty (26) suggests that individuals with non-A blood types have tumor cells with A-like antigens, which are perceived as foreign by the body and are thus more easily targeted for elimination. On the other hand, the antigens on tumor cells of individuals with blood type A closely resemble the A antigen in terms of molecular structure. Consequently, the immune system may struggle to recognize these tumor cells as a threat. This observation may partially explain why individuals with blood type A have a higher incidence of certain cancers compared to those with blood type O.

Our study is in its early stages, and we acknowledge certain limitations. In addition to the aforementioned “absence of disease-related blood group records,” we must also consider the impact of the diseases themselves. For instance, Helicobacter pylori (HP) infection has been suggested to have associations with blood groups (27), but routine HP screening is not part of the standard examination for hospitalized patients with hematologic malignancies. The presence of HP infection could potentially introduce interference in our statistical analysis. Furthermore, the control group’s population distribution inevitably includes individuals with other undisclosed diseases, which could confound our findings. Additionally, we recognize the age difference between our study cohort and the control group from Liu et al.’s (15) study, which may affect the comparability of the results. There is no perfect control cohort for this retrospective study. It is evident that a more comprehensive disease classification, along with larger-scale retrospective and prospective studies, as well as molecular level mechanistic investigations, are imperative. Moreover, our study primarily focused on Han Chinese patients, and the limited number of non-Han participants in the cohort made it unfeasible to analyze potential ethnic differences in blood group distribution. We hope that these efforts will serve as supplementary oversight in the public’s leukemia awareness and key population health examinations.

## 5 Conclusion

A significant decrease in blood type AB and an increase in blood type A prevalence among myeloid neoplasms were observed, shedding light on the connection between blood type and hematologic malignancies. Further research is needed to delve into the mechanisms that underlie this susceptibility to myeloid neoplasms as indicated by blood type.

## Data Availability

The raw data supporting the conclusions of this article will be made available by the authors, without undue reservation.
